# *Vagabond*: bond-based parametrization reduces overfitting for refinement of proteins

**DOI:** 10.1107/S2059798321000826

**Published:** 2021-03-30

**Authors:** Helen M. Ginn

**Affiliations:** a Division of Life Sciences, Diamond Light Source Ltd, Harwell Science and Innovation Campus, Didcot OX11 0DE, United Kingdom

**Keywords:** X-ray diffraction, refinement software, bonds, protein flexibility, models

## Abstract

Structural biology methods have delivered over 150 000 high-resolution structures of macromolecules, which have fundamentally altered our understanding of biology and our approach to developing new medicines. However, the description of molecular flexibility is instrinsically flawed and in almost all cases, regardless of the experimental method used for structure determination, there remains a strong overfitting bias during molecular model building and refinement. In the worst case this can lead to wholly incorrect structures and thus incorrect biological interpretations. Here, by reparametrizing the description of these complex structures in terms of bonds rather than atomic positions, and by modelling flexibility using a deterministic ensemble of structures, it is demonstrated that structures can be described using fewer parameters than in conventional refinement. The current implementation, applied to X-ray diffraction data, significantly reduces the extent of overfitting, allowing the experimental data to reveal more biological information in electron-density maps.

## Abstract 2

Reparametrizing the positions and flexibility of protein models for refinement against diffraction data results in a substantial reduction in the number of parameters. This produces maps that are less affected by model bias, which allow the maps to more faithfully reflect the true crystal contents and reveal more biological information.

## 1. Introduction   

Overfitting bias arises from the inherent mismatch between the paucity of experimental data and the complexity and size of biological macromolecules. It is exacerbated by the currently universal atomistic description of molecular structure, in which structures are described by atomic coordinates (*x*, *y*, *z*) combined with some indication of flexibility (typically the so-called *B* factor, which applies a Gaussian blur to the atomic position) (Adams *et al.*, 2010 ▸; Murshudov *et al.*, 2011 ▸; Bricogne *et al.*, 2017 ▸; Sheldrick, 2015 ▸). In the vast majority of cases this description requires a minimum of four parameters per atom to be refined against the experimental data. Additional parameters must be refined to permit more complex descriptions of macromolecular flexibility (anisotropic *B* factors, alternate conformations and rigid-body motions). In order to control overfitting, aspects of the model (bond lengths, angles, chirality, neighbouring *B* factors) are restrained using prior chemical knowledge, effectively increasing the number of observations (Konnert & Hendrickson, 1980 ▸), and a cross-validation metric (*i.e. R*_free_; Brünger, 1992 ▸) is used to check for undue overfitting. Despite these efforts, individual bond lengths in atomic models vary in a chemically unreasonable manner, frequently by over 0.01 Å from the expected values, in part owing to their systematic shortening due to anharmonic motion (Stuart & Phillips, 1985 ▸). In addition, quality metrics for the fit of the model to the data (for example *R* factors and real-space correlation coefficients) do not match the quality of the data. Thus, *R* factors generally stall 10–15% higher than the precision of the data itself (Holton *et al.*, 2014 ▸). A number of papers have addressed the damage to biological interpretation from these various pitfalls (Wlodawer *et al.*, 2017 ▸; Dauter *et al.*, 2014 ▸; Baker *et al.*, 2010 ▸; Jeffrey, 2009 ▸).

Early work in model refinement began in earnest in the 1980s (Hendrickson, 1985 ▸; Sussman, 1985 ▸; Tronrud *et al.*, 1987 ▸) and some researchers began to explore hybrid models of positional and bond-based models (Sussman, 1985 ▸; Oldfield, 2001 ▸). Although maximum likelihood does not require a certain parameterization scheme, the success of maximum-likelihood methods cemented the use of the atomistic models with which they were co-developed, and these models have become standard procedure. One major beneficial consequence of atomistic parametrization is that *R* values can be rapidly brought down from those calculated with an initial molecular-replacement solution, often 35–40%, to an interpretable structure with *R* values of around 20–25%, with some manual structural rearrangement required by the researcher. However, after decades of improvement in X-ray data quality, overfitting issues remain in model-building tools and in refinement software, suggesting that they cannot be addressed using the current atomistic modelling scheme. Therefore, I have developed an alternative approach that reduces the number of parameters by ∼60% and is freely available as a new macromolecular refinement package, *Vagabond*. This paper provides a proof of principle that changing the parametrization scheme can clarify difficult-to-interpret electron density, and that refinement is broadly stable across a random selection of entries from the PDB.

## 2. Methods   

**Figure 1:**
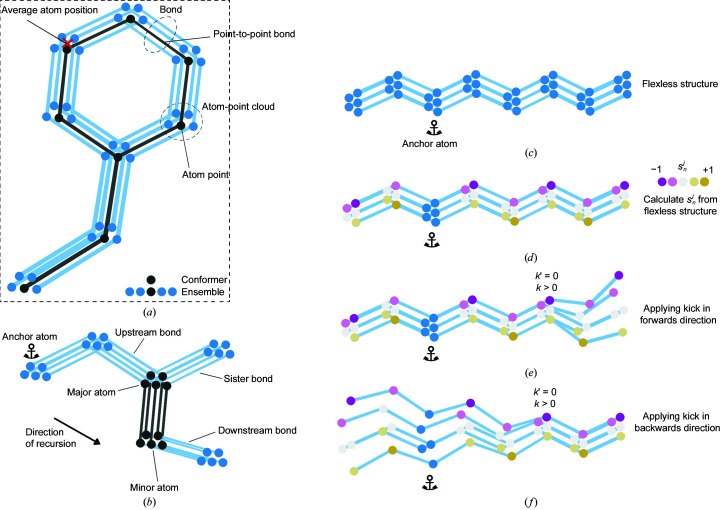
(*a*) Definition of terms relative to a given bond, drawn in black. (*b*) Separation and explanation of terms relating to the ensemble (**average atom position**, **bond** and **atom-point cloud**) and a single conformer in black (**point-to-point bond** and **atom point**). (*c*) The **flexless structure** incorporates no additional flexibility. (*d*) The **flexless structure** is used to calculate the value of 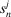 for each atom point, the dot product between 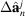 and 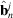, which can take a value of −1 to +1. These values are used to apply flex to the structure for a given bond by determining the relative magnitude of torsional deviation (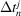 values). (*e*) This can be introduced via propagation through **downstream bonds** through simple recursion. (*f*) Flex can also be introduced only for **upstream bonds** by modifying the **atom-point cloud** of the **anchor atom** with simultaneous correction of the 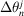 angles of the given bond to ensure no change in the **downstream bond** directions.

Defined terms used throughout this section are highlighted in bold and are defined in Table 1 ▸. Key terms are illustrated in Figs. 1 ▸(*a*) and 1 ▸(*b*). Symbols are defined in Table 2 ▸. This section is divided into a description of the model and the calculation of electron density, followed by the refinement method.

### 2.1. Generation of an initial bond-based model   

Atoms from a PDB file are loaded into memory and the connectivity between atoms is calculated from the sequence, residue numbers and prior information of residue connectivity. Atoms listed under the heteroatom (HETATM) category are included using their original isotropic or anisotropic *B*-factor definition, and are not currently remodelled by the *Vagabond* refinement process. An **anchor atom** is chosen per amino-acid chain, from which the rest of the same chain will be generated. There is no clear dependency of the outcome of the refinement on the choice of the **anchor atom**, and so as a conservative choice, to avoid the extremes of the model, *Vagabond* defaults to the backbone N atom of the residue closest to the centre of mass of the chain. Backbone N atoms are not considered if they have an alternative conformation as defined in the PDB file. The initial positions from the PDB file are held in memory, and are hereby referred to as **original positions**. Canonical bond lengths, angles (Engh & Huber, 2001 ▸) and chirality are imposed on the model, whereas initial torsion angles are calculated for appropriate atoms from the **original positions** relating each set of four sequential atoms. A number of fixed torsion angles can also be enforced (such as within the tyrosine ring). **Downstream bonds** connect the **anchor atom** to the N- and C-termini. At branch points, such as β carbons, carbonyl O atoms and all H atoms, multiple **downstream bonds** constitute **sister bonds** (Fig. 1 ▸ *b*). The branched bonds off the main chain are all affected by the torsion angles along the main chain. The bonds for these branches are related to the main-chain **upstream** and **sister bonds** by two bond angles. H atoms are regenerated at appropriate torsion angles at a bond length of 0.968 Å.

### 2.2. **Atom-point cloud** generation   

A cloud of points for the **anchor atom** is generated by taking the desired number of **conformers** (*J*; default 120) and arranging them into ten concentric spherical surfaces centred around the **original position** of the **anchor atom** (Fig. 1 ▸ *b*), populated by a number of points proportional to the surface area of each layer. The outermost layer has a radius ω, which has an initial value of 0.356 Å and may be changed during refinement. The **atom points** on each layer are arranged using a Fibonacci lattice (González, 2010 ▸) to produce a roughly uniform distribution on each layer.

### 2.3. Calculation of the **flexless structure**   

All sequentially bonded **atom-point clouds** are recursively placed towards both the N- and C-termini, according to their relationship to the previous three **atom-point clouds** using only the bond lengths, angles (θ_*n*_) and mean torsion angles (*t*_*n*_) for each atom *n*. Each **atom point** is uniquely paired with an **atom point** in each of the sequentially bonded **atom-point clouds**. This calculation does not include any description of flexibility, leading to a simple duplication of the initial **atom-point cloud** at every **atom average position**. The resulting **ensemble** is referred to as the **flexless structure** (Fig. 1 ▸ *c*).

### 2.4. Calculation of whole-molecule movements   

The set of whole-molecule movements provide rules upon which to apply rotations, translations and rotation–translation coupling, and are applied to the **flexless structure** before any other contributions from intramolecular flexibility. The description is inspired by TLS (Winn *et al.*, 2001 ▸), but is not equivalent, as it defines an explicit and unique combination of rotations and translations for each **atom point** of the **anchor atom**, while TLS describes the average motion derived from rotations and translations from a mean position with a variable level of correlation.

The translation is conferred by a symmetrical tensor **T** with six refineable parameters. The initial value of **T** is set to the identity matrix. To apply translations to each **atom point**, singular value decomposition (SVD) is performed on this matrix, 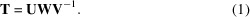



As **T** is invertible, the matrix **U** contains the eigenvectors of this matrix in each column and the matrix **W** contains the eigenvalues. For each **atom point** in the **atom-point cloud** of the **anchored atom** in the **flexless structure**, an offset per **atom point** 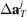 is calculated (see Table 2 ▸), 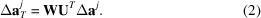



Any number of additional screw motions (a default of three, but user-changeable) are applied to these updated positions. Each comprises a rotation and a rotation-dependent translation. For each screw motion, a three-dimensional vector **r** defines the rotation axis and angle, and a three-dimensional vector **w** defines a rotation-dependent translation, of which the third vector component is set to zero. This constitutes five parameters per screw motion.

Let *d*_*j*_ be the dot product between 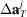 and **r** for every **atom point** *j*. A rotation matrix **D**_*j*_ is constructed to rotate by an angle of *d*_*j*_ around the unit vector for **r**, 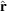. The corresponding **point-to-point bond** vector is multiplied by **D**_*j*_. This has the effect of ‘fanning out’ the **conformers** in the **ensemble**. Additionally, a basis matrix **S** is calculated as in (3) to produce an additional translational offset per **atom point**:
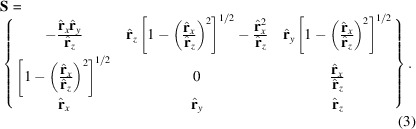
This produces a matrix of orthogonal basis vectors. The vector **Sw** produces a translational offset in the plane perpendicular to 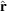, which is then applied as in (4). The magnitude of the second term in (4) is proportional to the value of *d*_*j*_ and is therefore a rotation-dependent translation. Here, the summation symbol indicates the sequential application of each pair of **w** and **r** parameters. 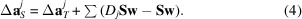



The translated positions for each **atom point** of the **anchor atom** are then calculated as 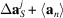. The application of whole-molecule movements only changes the values of the **atom points** and **point-to-point bonds** of the **anchor atom** directly. The global motions are then propagated through the rest of the polypeptide chain. Certain intramolecular movements are further applied to the **atom-point cloud** of the **anchor atom** in the next section.

### 2.5. Calculation of intramolecular flexibility   

This section describes how torsion angles are allowed to vary between **conformers** of an **ensemble**, describing how the magnitude of the variation is defined and also the use of two variable angles to modify the axis upon which this variation is applied. Kick parameters associated with a **bond** confer flexibility through deviation of the torsion angles between the **point-to-point bonds** controlling the distribution of the **atom points** of the **minor atom** of the bond. The **flexless structure** is used to determine how the kicks will be applied to each **point-to-point bond** torsion angle. The plane *P* intersects the average vector of each bond 〈**b**_*n*_〉 and the previous bond 〈**b**_*n*−1_〉. The unit vector 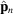 is perpendicular to plane *P*. A rotation matrix **M**_*n*_ is defined in terms of two angles, α_*n*_ and β_*n*_. This custom rotation definition rotates around the ***x*** axis by an angle of α. The multiplication of this rotation matrix with a vector in the direction of the ***y*** axis will produce vector **y**′. A rotation angle of β is then applied around the *y*′ axis. A single matrix describing the product of these sequential rotations is described in (5):
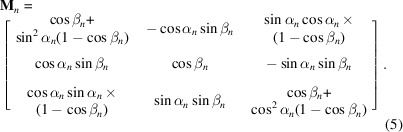



This definition is used as this matrix can produce a custom eigenvector with two angles without the risk of gimbal lock. The default values of α_*n*_ and β_*n*_ are zero, and therefore produce no effect, but may optionally be refined. For each **atom point**, a vector can be used to describe the displacement of the **atom point** from the **average atom position**, 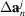. The scalar 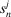 is the sine of the angle between the vectors 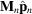 and 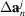 per point in the atom-point cloud. These are only ever calculated from the information in the **flexless structure** and are then stored for future use (Fig. 1 ▸ *d*).

Each C^α^ **atom-point cloud** in the structure may have two kicks associated with it. Flexes onwards from the N-terminal and C-terminal sides of the **anchor atom** are considered to always be in the forward direction, with an associated kick parameter *k*_*n*_. The sign of *k*_*n*_ is interchangeable with the direction of the custom eigenvector generated by α_*n*_ and β_*n*_. The reverse direction has a kick parameter *k*′_*n*_. For forward-direction kicks, the torsion-angle deviation 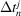 per **atom point** is calculated as the product of its pre-calculated sine angles and both of the associated kick parameters of the **bond**, 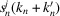. These deviations will then be propagated to the downstream terminus on calculation of the **downstream bonds** (Fig. 1 ▸ *e*). Reverse-direction propagation is conceptually different as it involves breaking the recursion. In this case, for each **atom point** of the C^α^ atom, an appropriate rotation is applied to the **atom point** of the **anchor atom** in the same **conformer**. This rotation has an angle of 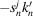 around the corresponding **point-to-point bond** vector 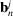 that has the C^α^ atom as its **minor atom**, and the centre of rotation is the C^α^ atom. Note that the 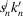 term in the forward direction cancels out the 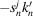 term in the reverse direction to terminate flexibility in the **downstream bonds** (Fig. 1 ▸ *f*). The order in which these kicks are applied to the chain runs from N-terminus to C-terminus; the order choice is arbitrary but must be maintained for correct recalculation of the structure.

### 2.6. Calculation of real-space structure segments   

In order to calculate a portion of real-space structure from the model, electron density is generated on a grid composed of voxels with cubic morphology, known as a **real-space structure segment**. The method described here differs from the usual method (Ten Eyck, 1977 ▸) as this method is computationally very slow for *Vagabond* models, and the lack of individual atomic *B* factors provides new opportunities for calculating maps with efficiency. For a nominal resolution *d*, the cube voxel length is *d*/4. The nominal resolution is taken as the highest resolution recorded reflection in the reflection list. This cube is capped at a maximum length of 0.8 Å. A cuboid grid is generated by choosing a number of voxels in each dimension capable of encompassing the calculated volume of interest (the bounding box containing the full **ensemble**) and an additional margin of 2 Å on each cuboid face.

Atoms to be inserted into the calculated regions are grouped by element and treated separately. Atoms currently fall into two categories. Some employ a *B*-factor-based model. These are recorded as HETATMs in the original PDB entry and have not been remodelled in the current study. The second category are those generated by *Vagabond* as detailed above. In the former case, the anisotropic or isotropic *B* factor associated with the atom produces an atomic distribution, which is first calculated in reciprocal space and then transformed to real space. This is added to the electron-density map at the appropriate grid points near the **atom average position** using 11-point interpolation as implemented in the *General Averaging Program* (*GAP*; Ginn & Stuart, 2016 ▸). For the *Vagabond* atoms, the **atom-point clouds** are added individually to the map. Each **atom point** will land between eight grid points and contribute some density to each. Eight smaller fractional cuboids are delineated by drawing dividing planes passing through the **atom points** orthogonal to each principal axis. Each voxel vertex is then assigned a proportional contribution according to the volume of the diagonally opposed fractional cuboid. Although this is not necessarily in the spirit of discrete sampling for Fourier transforms, it has the advantage of maintaining the total electron density per atom and tends towards similar behaviour with sufficiently small grid spacing. The addition of a large number of interpolated samples for each **atom point** at slightly different positions smoothens the map, protecting against Fourier truncation errors in a similar manner to applying a real-space *B* factor. Once all the atoms of a given element have been placed in the map, the map is transformed to reciprocal space. The reciprocal-space scattering factors of the given element from Table 6.1.1.1 in *International Tables for Crystallography* Volume C (Brown *et al.*, 2006 ▸) are used to calculate the appropriate amplitude for each structure factor. These two structure-factor lists are multiplied and then transformed to produce a real-space map of all atoms of a given element type. The partial maps for every element are then summed together to produce the final calculated real-space map.

Note that the reciprocal-space transformation of this map does not correspond to *F*_model_. It is generated on a cubic grid spacing with an unrelated origin and different dimensions to the crystallographic unit cell, and may also only contain a region of interest rather than the entire structure. In the special case where the region of interest encompasses the entire modelled content of the asymmetric unit, this is referred to as the **asymmetric unit structure**.

### 2.7. Calculation of asymmetric unit structure factors   

On the way to producing *F*_calc_ structure factors, the **asymmetric unit structure** is remapped onto a **unit-cell grid**, which has a voxel grid spacing consistent with the unit-cell dimensions of the crystal. The voxel dimensions for the **unit-cell grid** are also chosen to sample the contents at a *d*/4 spacing. The contents of the **asymmetric unit structure**, as generated above, are added into the appropriate positions of the **unit-cell grid**. By looping through the voxel vertices within the bounding volume in the **unit-cell grid**, the appropriate fractional voxel position is calculated from the **asymmetric unit structure**. The density value at this position is estimated via 11-point interpolation (Ginn & Stuart, 2016 ▸) and added to the **unit-cell grid**. *F*_expl_asu_ are the structure factors of defined atoms belonging to one asymmetric unit, calculated by taking the Fourier transform of this final real-space map.

### 2.8. Calculation of bulk-solvent model   

The solvent mask is calculated for the **unit-cell grid**. A separate solvent mask is calculated from each **conformer** in the **ensemble**. Protein voxels are set to zero and solvent voxels are set to a nominal positive density of 1.0 for the mask of each **conformer**, and then all *J* solvent masks are averaged. Due to the lack of an *F*_000_ measurement and a separate solvent scale factor, the exact value of the nominal positive density does not matter. To create each *j*th solvent mask, as previously determined (Jiang & Brünger, 1994 ▸), non-H **atom points** of the *j*th **conformer** are used to mask out the voxel positions within the specific radii defined previously (Jiang & Brünger, 1994 ▸) around their designated position by element or atom type. The solvent mask is then expanded by switching all model voxels that occur at most 0.4 Å away from a solvent voxel to solvent. The final stage removes small internal strips of solvent density which may have been retained inside the protein interior by setting solvent voxels to protein if they occur in strips of less than 2.0 Å along each crystallographic axis.

In order to make multi-conformer solvent-mask calculations feasible for large values of *J*, in terms of both memory consumption and computation time, solvent masks are calculated using bitwise operators over 32 bits of memory, allowing 16 masks to be calculated concurrently without overwhelming memory consumption and with an increase in computation speed within a single CPU thread. *F*_solv_asu_ are the structure factors for the average bulk solvent belonging to one asymmetric unit, calculated by taking the Fourier transform of this final real-space map.

### 2.9. Application of space-group symmetry   

The space-group operators corresponding to those listed in the original PDB header file are applied by cumulatively adding the symmetry-transformed complex Fourier coefficients of each structure factor to its symmetry-related Miller indices in reciprocal space. Reciprocal-space addition is preferred to avoid interpolation errors for space groups where symmetry operations would not correspond to integral voxel spacing in real space. This is performed separately for both *F*_solv_asu_, to produce *F*_solvent_, and *F*_expl_asu_, to produce *F*_explicit_.

### 2.10. Calculation of *F*_calc_ structure factors   

The *F*_solvent_ structure factors are scaled using two parameters, an absolute scale *k* and a *B* factor *B*, such that 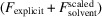 has the highest correlation coefficient when compared with *F*_observed_,
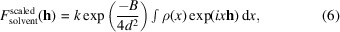
where **h** is the reciprocal-lattice point, 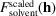 is the structure factor for a reciprocal-lattice point at vector **h**, *d* is the reciprocal of the magnitude of **h**, and ρ(*x*) is the density of the solvent at fractional real-space position *x*. The values of the two parameters are re-refined during each recalculation of the weighted electron-density map using the simplex method of gradient descent for 100 cycles (Nelder & Mead, 1965 ▸), using initial step sizes of *k* = 0.4 and *B* = 40 Å^2^, with initial values of 0 and 40 Å^2^, respectively. The addition of the two sets of structure factors according to these chosen scales, 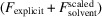, produces the set of *F*_calc_ structure factors.

### 2.11. Calculation of weighted electron-density maps   

*F*_calc_ is scaled to *F*_observed_ by multiplying the *F*_calc_ values so that the average amplitude is equal to that for *F*_observed_ in 20 equal-volume resolution bins in reciprocal space. A weighted electron-density map is then generated using a newly devised weighting scheme to generate the target for real-space refinement. This relies on the principle that downweighting reflections by multiplying the amplitude by a weighting factor is a simulation of uncertainty in phase angle. A standard error, *s*_*k*_, is calculated for each reflection *k* equal to the ratio between σ(*F*_observed,*k*_) and *F*_observed,*k*_. In this weighting scheme, a Gaussian distribution of phase angles is determined for each acentric reflection. A downweighting term *d*_*k*_ is defined as 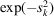 and lies between 0 and 1. An appropriate standard deviation for a Gaussian-distributed set of phase angles (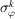) must be calculated. Considering a circle of radius 1, inclusion of the radial point at each phase angle φ at its relative weight as determined by the Gaussian function 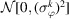 produces a weighted arc. The centroid of this weighted arc should have a radius of *d*_*k*_. This centroid was calculated for values of 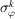 between 0 and 6.27 radians (≈2π) in steps of 0.01, covering values of *d*_*k*_ between 0.0308 and 1. This is then used as a lookup table in reverse for establishing values of 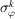 for a given value of *d*_*k*_. The maximum value of 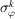 used is 6.27 radians. Each reflection is introduced into a unit-cell grid in reciprocal space at an amplitude of 2*F*_o_ − *F*_c_ in this current implementation. 25 separate Fourier transforms are calculated, where each reflection is included at its calculated phase plus an incremented phase shift ranging from −2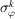 to +2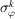 in 25 equal steps, and then multiplied by its Gaussian-derived weight. These are transformed to real space and summed to produce a new weighted density map for further refinement. Reflections included in the *R*_free_ set are omitted from this calculation. By including a range of phases, this allows interference which would occur between structure factors due to the uncertainty in their phase, as the sum of the 25 contributions for each structure factor is no longer truly harmonic.

### 2.12. *Vagabond* refinement engine   

Refinement is split into several modes: positional refinement against the original PDB file (mode 1), main-chain flexibility macrocycles comprising the refinement of one whole molecule (mode 2a) and intramolecular flexibility (mode 2b) against the electron density, and a final mode of side-chain refinement against the electron density (mode 3). After each stage of whole-molecule refinement, if the *R*_work_ value increases the structure is reverted to the parameters in the previous cycle. This allows compensation to be made for more motion using intramolecular movement instead. If the value of *R*_work_ is increased after three cycles of intramolecular refinement, the overall flexibility is incrementally reduced by a 10% reduction in the parameter ω for each polymer chain for as long as *R*_work_ continues to decrease due to the contraction. Additionally, before the refinement of side-chain motion, the algorithm reverts to the cycle with the lowest value of *R*_work_. If side-chain refinement causes an increase in *R*_work_ then these parameter changes are also reverted.

### 2.13. Positional refinement (mode 1)   

**Figure 2:**
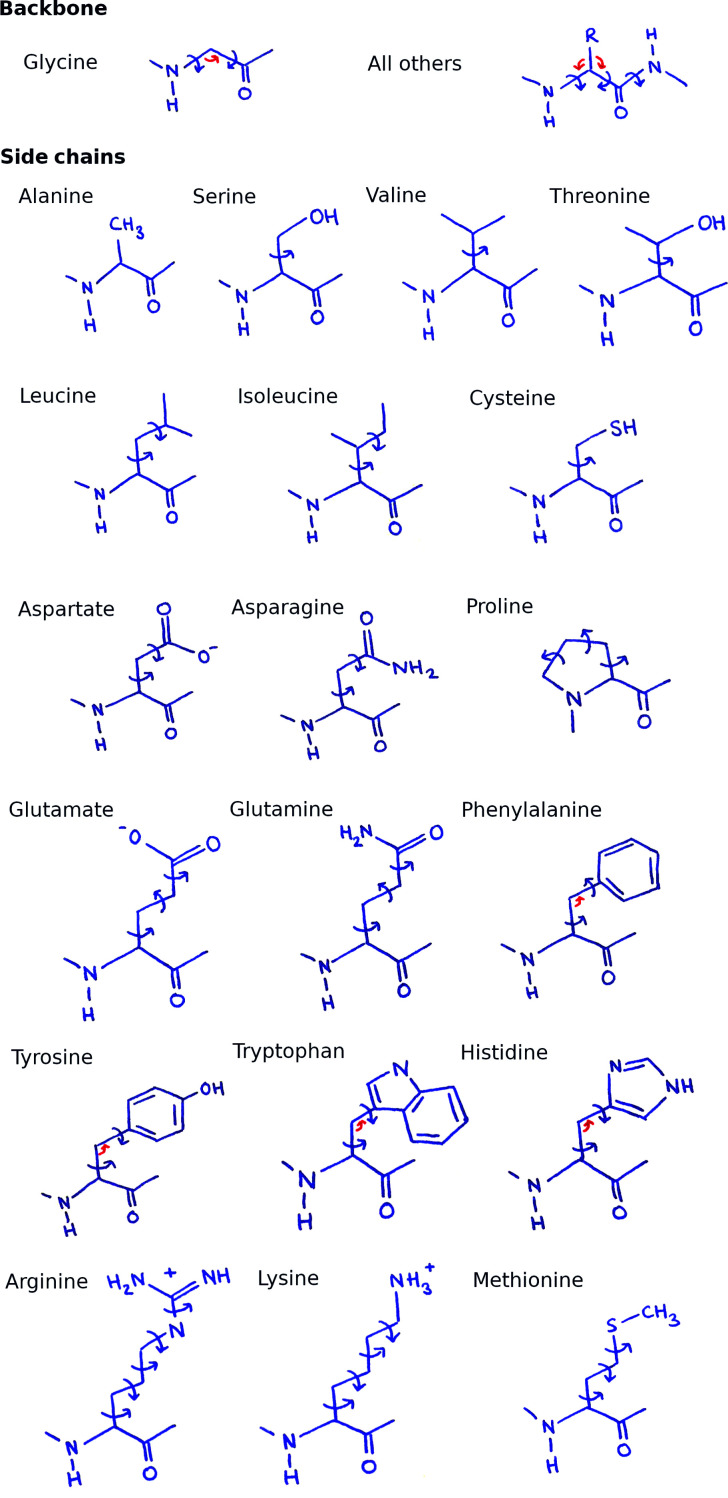
Refined torsion-angle (blue ink) and bond-angle parameters (red ink) marked on the backbone and additional parameters for each amino acid. All marked torsion angles also have refined kick parameters apart from proline side-chain bonds and the peptide bond.

Refined torsion parameters and bond angles are shown for the backbone and each amino acid in Fig. 2 ▸. The simplex method of gradient descent (Nelder & Mead, 1965 ▸) is used to minimize the unconstrained torsion-angle parameters of the bond-based model to match the mean absolute difference between the **original positions** reported in the original PDB file and the **average atom positions** calculated from the *Vagabond* model. This removes the initial error propagated through the chain due to imperfect initial torsion-angle estimations. Torsion angles around four sequential backbone bonds are considered in one simplex minimization run, extending from the **anchor atom** to the N-terminus and then from the **anchor atom** to the C-terminus. Atoms considered in evaluation of the target function include all major and non-H **minor atoms** of refined bonds, as well as non-H **minor atoms** of any **sister bonds**. This therefore includes the carbonyl O and C^β^ atoms. After minimization of four bonds, the starting bond is advanced by one and the process is repeated until the chain ends. Convergence is considered to be no shift of torsion angles beyond 0.005° or when a maximum of 60 simplex descent steps is reached. After all backbone atoms which may affect a given C^β^ atom have been refined, refinement of the torsion angles in the side chains is allowed to proceed. This scheme therefore prioritizes fitting the path of the backbone over the side chain to the **original positions**. This is carried out five times before calculation of the first electron-density map.

Two of these cycles are carried out without refining bond angles and with a torsion step size of 2°. A further three cycles allow bond-angle refinement for all C^β^ atoms and C^γ^ atoms of aromatic residues with starting torsion-angle and bond-angle step sizes of 0.2°. This therefore prioritizes the reduction of errors with torsion angles before introducing bond angles into refinement.

### 2.14. Correlation coefficients between weighted and calculated electron density   

When a portion of the structure is selected as a target for a number of cycles of refinement, several aspects of the **real-space structure segment** are calculated only once and reused, including the map segment position and dimensions. The exact choice of **real-space structure segment** are clarified in the following sections. A number of atoms may be selected for refinement (active atoms). The **real-space structure segment** containing this portion of the structure will encompass additional atoms (surrounding atoms). The **real-space structure segment** for the surrounding atoms is calculated once in a separate map in memory and added to the calculated density on each cycle of refinement. On each update of the model, the set of **atom-point clouds** for the active atoms are recalculated and the **real-space structure segment** is recalculated. This segment will fall in a region of the weighted electron density. In order to compare the calculated and the weighted structures, a list of voxels is prepared in the coordinate system of the **unit-cell grid** which map onto the full volume of the **real-space structure segment**. Each weighted density grid point will then fall upon a fractional voxel coordinate in the **real-space structure segment**. The calculated density is then estimated via 11-point interpolation, both for the active-atom map alone and for the full map corresponding to the addition of the active-atom map and the surrounding-atom map. A Pearson-weighted correlation coefficient is calculated comparing the weighted density values with the full calculated map density values, and the weights are for the active-atom map values alone. This target function is then maximized by changing various model parameters.

### 2.15. Whole-molecule refinement (mode 2a)   

Each cycle of whole-molecule refinement will cycle through each polymer chain in the asymmetric unit, choosing the limiting **real-space structure segment** which contains all starting **atom point** positions of the polymer backbone with a 3 Å margin. The eventual target is to have three screw motions defined, which are introduced one by one during the first macrocycle. Initially only a single screw motion is introduced. Each of the following refinement protocols occur using the simplex method of gradient descent for a maximum of 100 cycles. The target function is the correlation coefficient for the set of active atoms encompassing the backbone atoms of the given polymer chain, including C^β^ atoms.

For an introduced screw motion, a new rotation direction is determined by testing 31 roughly equally spaced directions generated using a Fibonacci lattice. This is followed by refinement of the three parameters of **r**, and then a separate refinement of the two parameters of **w** for all existing screw motions. In the first cycle, second and third screw motions are introduced directly afterwards. Finally, the six components of the translation tensor **T** are refined. In subsequent macrocycles no additional screw motions are introduced, but the **r**, **w** and **T** parameters each receive one round of refinement against the target function.

### 2.16. Intramolecular flexibility refinement (mode 2b)   

Using the same **real-space structure segment** as in mode (2a), **bonds** with similar effects are grouped for refinement in batches. To determine the batches, each **bond** receives a similarity score with every other **bond** under the following regime. For every pair of **bonds** in a polymer, each C^α^ atom is considered in turn. For each **atom point** of the C^α^ atom, the direction and magnitude of a rotation around each of the **point-to-point bonds** in the **bond** pair is known. The similarity of the directions and magnitudes of the potential rotation vectors are scored against one another by the cosine of the angle between them, multiplied by the smaller ratio of the two magnitudes. This score is multiplied by both of the 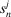 values associated with the **atom points** of the **minor atoms** of the two **bonds**. These scores are unity in the case of a perfect match. These are summed over all **atom points** of a C^α^ atom and are further summed over the set of C^α^ atoms of the same polymer. If two bonds (*p*, *q*) have a score of *x*, the bonds (*q*, *p*) have a score of − *x*. The value of (*p*, *p*) is set to zero instead of unity. SVD is performed on the full set of **bond–bond** relationships. Either the top ten vectors, or the number required to cover 50% of the total sum of decomposed vector lengths, whichever is larger, are considered for refinement. For each cluster, the *k* and *k*′ parameters can be varied independently, thereby producing a minimum of 20 refined parameters at this stage. For each round of refinement, the relative contribution of a cluster to the values of *k* and *k*′ associated with each bond are calculated and summed over all clusters for each bond. The target function, as for whole-molecule refinement, is the correlation coefficient for the active atoms encompassing the backbone and C^β^ angles of the polymer chain. This is repeated once for each polymer chain.

### 2.17. Side-chain refinement (mode 3)   

Every side chain undergoes refinement in turn. The chosen **real-space structure segment** bounds all atoms in the amino acid being refined with a 3 Å margin. For each side chain, the target function is the correlation coefficient against the **real-space structure segment** for the entire monomer, including the backbone, as the set of active atoms. The parameters refined are the side-chain torsion and refinable bond angles (step size of 0.1°), kick parameters (a dimensionless step size of 0.5) and α and β angles (step size of 30°).

### 2.18. Choice of PDB entries from the Protein Data Bank   

111 803 structures were marked as suitable for refinement; they were required to contain X-ray data and no nucleic acid polymer chains. Resolution was limited to between 1.2 and 3.5 Å. Of these, every hundredth structure was taken for benchmarking. Structures were removed if they contained alternative conformers of varying sequences, and other edge cases were removed (such as incomplete amino-acid backbones). This left a total of 920 structures in the benchmarking set.

## 3. Results   

**Figure 3:**
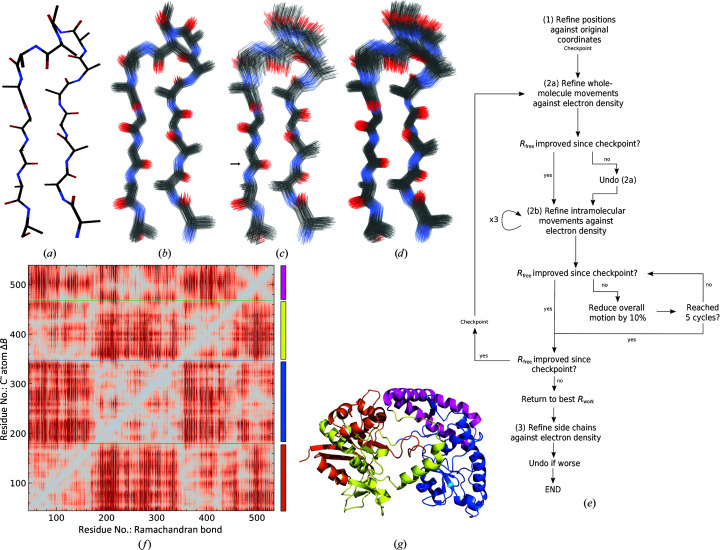
(*a*) 15 residues in a polyalanine β-sheet with the β-loop showing average positions described by 45 backbone torsion angles. (*b*) The array of *n* individual conformers, from which the average in (*a*) was derived, is shown here with no additional flexibility (*n* = 120). (*c*) The array in (*b*) has been modified by using an applied equal forward and backwards kick (one parameter, target C^α^ marked with an arrow) propagated in both directions to introduce flexibility in the β-loop, producing a variable torsion angle in the two bonds linked to the C^α^ atom between elements of the array (*n* = 120). (*d*) The same model as in (*c*) but with increased sampling (*n* = 300). Increasing the sampling does not increase the number of parameters. (*e*) Workflow for the fitting procedure in *Vagabond*. (*f*) The effect of small perturbation of the Ramachandran bond torsion angle against the change in r.m.s.d. of each C^α^ atom-point cloud for PDB entry 5g5y chain *A*. Red denotes an increase, grey no change and blue a decrease. Lines denote visually separable domains. (*g*) Marked boundaries from (*f*) on the polypeptide chain, shown as orange → blue → yellow → magenta, drawn with *PyMOL* (DeLano, 2002 ▸).

*Vagabond* describes a macromolecular structure as a series of deterministically related conformers, collectively forming an ensemble, with one ensemble calculated for each protein chain in the model. Each conformer comprises a network of bonds connecting atoms (Rice & Brünger, 1994 ▸). The construction of the ensemble starts from a single anchored atom, which is defined with an absolute position within the crystallographic unit cell. Each atom is described by an atom-point cloud (Figs. 3 ▸ *a* and 3 ▸ *b*). Applying a forwards and backwards kick to a C^α^ atom spreads the distribution of every atom accordingly (Fig. 3 ▸ *c*). The number of atom points calculated in each atom-point cloud determines the level of sampling of the statistical distribution, but does not affect the overall flexibility or the number of parameters used to describe the model (Fig. 3 ▸ *d*). This description of flexibility allows a bulk-solvent model comprising the sum of all solvent distributions calculated for each explicit individual conformer. The workflow for refinement in *Vagabond* is summarized in Fig. 3 ▸(*e*). For the present purposes, models from the endpoint of refinement using current state-of-the-art atomistic refinement serve as the input to generate a starting model for *Vagabond*. The vagabond GUI requires two inputs: a reflection list supplied as an MTZ file and atomic coordinates supplied as a PDB file. Output files from *Vagabond* are weighted structure factors (MTZ file) for the electron density, summary output coordinates (PDB files, average and ensemble) and a *Vagabond* (.vbond) file format to store the bond-based model description.

The following analysis will discuss model bias. In this paper, the term ‘model bias’ is used to refer to the total observable effect of model-derived errors on the electron density. This comprises overfitting bias, noise bias and phase bias. Overfitting bias is caused by errors in the model definition itself and/or insufficient data to support its refinement. Noise bias involves misfitting a model to noise in the data, and can also exacerbate overfitting bias. Phase bias is the intrinsic unavoidable effect of using estimated phases in the Fourier transform from an incorrect model, leading to a bias in the electron density towards the incorrect model. This phase bias cannot be avoided in *Vagabond*, but model bias can be reduced through a reduction of the overfitting bias.

Describing structures through torsion-angle-mediated flexibility facilitates the subdivision of a protein into constituent domains and subdomains. Fig. 3 ▸(*f*) shows the effect of introducing equal backwards and forwards flexibility in each C^α^ backbone torsion angle on the change in r.m.s.d. for each C^α^ atom-point cloud for the *Streptococcus pneumoniae* ABC-transporter protein FusA (Culurgioni *et al.*, 2017 ▸). This can be visually segmented into regions corresponding to domains (and subdomains) within the polypeptide chain, each of which has been given distinct colours (Fig. 3 ▸ *g*). This simple analysis should also facilitate the automated definition of appropriate domains for TLS refinement.

**Figure 4:**
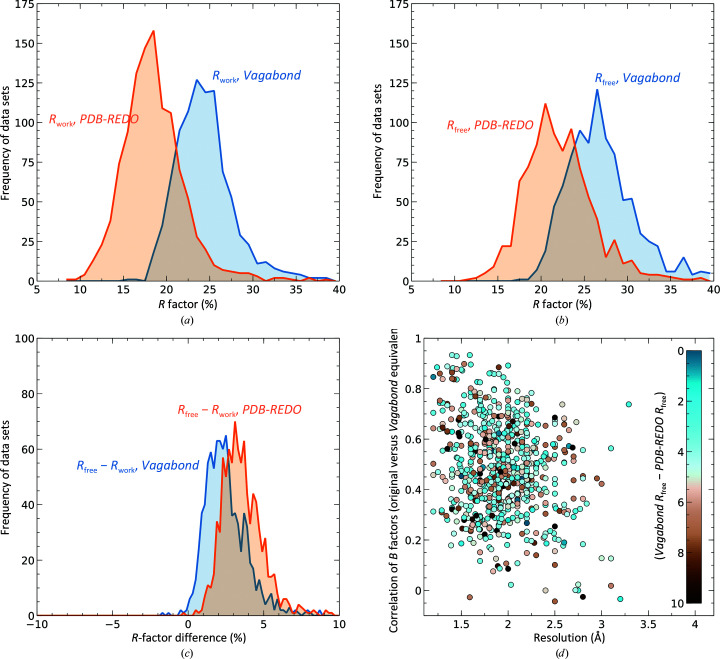
(*a*) Comparison of *R*_work_ values on a reduced set of 920 entries as reported by *PDB-REDO* and from *Vagabond* refinement. (*b*) Similar histogram derived from *R*_free_ values from *PDB-REDO* and *Vagabond*. (*c*) Comparison of the *R*-factor gap (*R*_free_ − *R*_work_) between *PDB-REDO* and *Vagabond*. (*d*) Correlation between *Vagabond*-derived and original *B* factors across the resolution range for the benchmark set with a colour indication of the discrepancy between the original and the *Vagabond* *R*_free_. 224 structures for which *PDB-REDO* did not refine individual *B* factors have been removed from the comparison.

To objectively investigate the applicability of *Vagabond*, 920 PDB entries were used as a benchmark set (Section 2). The data and the corresponding models were taken from the *PDB-REDO* server (Joosten *et al.*, 2014 ▸), which generates optimized structures using the latest algorithms and a state-of-the-art refinement engine. *Vagabond* was run with default settings (no user-defined per-data-set parameters) across this data set to produce mean *R* factors of *R*_work_ = 24.5% and *R*_free_ = 27.1%, which are compared in Figs. 4 ▸(*a*) and 4 ▸(*b*). These are higher than the original mean *R* factors from *PDB-REDO* (*R*_work_ = 18.5%, *R*_free_ = 22.2%) but exhibit a substantial reduction in the *R*_work_/*R*_free_ gap (Fig. 4 ▸ *c*), which is a generally accepted metric for the degree of overfitting (Brünger, 1992 ▸). This reduction remains where *Vagabond* achieves comparable *R*_free_ values. Thus, where the *Vagabond*-derived *R*_free_ values are within 4% of the original value, the *R*_work_/*R*_free_ gap is 2.5%, compared with the original value of 3.9%. Structures at a higher resolution than 1.5 Å have the largest discrepancy between the original and *Vagabond* *R*_free_ values (6.7%). The discrepancy is smaller for the remainder of the structures (4.8%). The *B*-factor equivalents derived from each C^α^ atom-point cloud in a structure sometimes show very high correlation with the original *B* factors in the PDB file (Fig. 4 ▸ *d*). For 224 structures (average resolution 2.7 Å), *PDB-REDO* did not attempt to model individual *B* factors and these results are not shown. In several cases the maps from *Vagabond* show additional information beyond that gained from conventional refinement. Two examples are detailed below.

**Figure 5:**
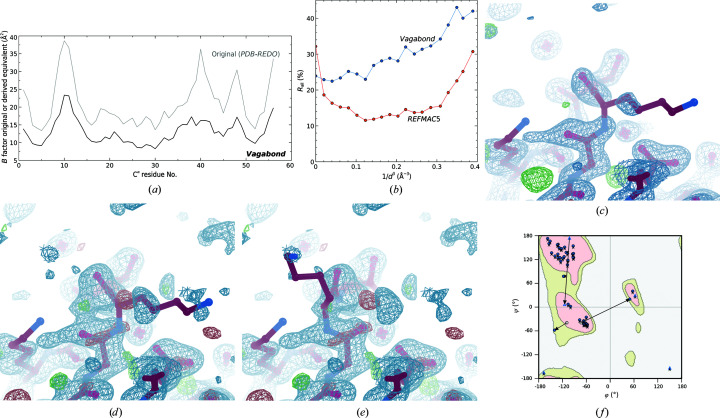
(*a*) Plot showing the *B* factor from the original PDB file (grey) and the derived equivalent *B* factor from the *Vagabond* structure (black) per C^α^ atom. (*b*) Final *R* factors per resolution bin for *REFMAC*5 and *Vagabond* for the occupancy-corrected structure. Apart from an improvement at resolutions lower than 3.7 Å, *Vagabond* gives higher *R* factors throughout the rest of the resolution ranges. (*c*) After correcting occupancy values, density from the *PDB-REDO* model refined using *REFMAC*5 was overlaid on the structure (2*mF*_o_ − *DF*_c_ 1σ, *F*_o_ − *F*_c_ 3σ). (*d*) The *PDB-REDO* model refined in *Vagabond* produces new electron density, which is overlaid on the original model (2*mF*_o_ − *DF*_c_, 1σ; *F*_o_ − *F*_c_, 3σ). (*e*) Refitting of the model to *Vagabond*-derived electron density in *Coot* showing improved backbone fit against electron density (2*F*_o_ − *F*_c_ plus phase spread, Section 2, 1σ; *F*_o_ − *F*_c_ plus phase spread, 3σ). (*c*), (*d*) and (*e*) were drawn with *Coot* (Emsley *et al.*, 2010 ▸) and *Raster*3*D* (Merritt & Bacon, 1997 ▸). (*f*) A Kleywegt plot drawn using *Coot* shows large movement of three residues, of which Lys10 switches from one minimum in the Ramachandran plot to another.

The first example shows that even at high resolution, atomistic refinement overfitting can obscure electron-density maps. The small immunoglobulin-binding domain Gβ1 is a frequent target for *in vitro* evolution and computational design in protein engineering (Wunderlich *et al.*, 2007 ▸; Ross *et al.*, 2001 ▸; He *et al.*, 2005 ▸; Thoms *et al.*, 2009 ▸; Reinert & Horne, 2014 ▸; Tavenor *et al.*, 2016 ▸). PDB entry 2on8 is an engineered mutant solved at 1.35 Å resolution (Wunderlich *et al.*, 2007 ▸). The original deposition had many lysine side-chain atoms reduced to an occupancy of 0.01; after correction and atomistic refinement with anisotropic *B* factors, *R*_work_ and *R*_free_ reached 16.0% and 20.7%, respectively. Refinement with *Vagabond* produced similar derived relative *B* factors to the original model (Fig. 5 ▸ *a*), with the exception of an absolute *B*-factor offset and a reduced *B*-factor peaks in loops. This resulted in higher *R* factors, except in the lowest resolution bin (Fig. 5 ▸ *b*), but with a smaller *R*_work_/*R*_free_ gap (24.7% and 26.3%, respectively). *Vagabond* clarified the original density (Fig. 4 ▸ *c*) for Lys10 to show a flipped peptide bond (Fig. 5 ▸ *d*), which was remodelled in *Coot* (Fig. 5 ▸ *e*; Emsley *et al.*, 2010 ▸) to occupy a different valid minimum in the Ramachandran plot (Fig. 5 ▸ *f*). This also explains the reduced peak in *B* factors in the loop region including Lys10, as this has been modelled with a single conformation in atomistic refinement, whereas the true structure is likely to have a major occupancy for the flipped Lys10 peptide bond and minor occupancy for the unflipped peptide bond. The *B* factors from the original atomistic distribution would be likely to inflate under these circumstances in order to attempt to compensate for the assignment of full occupancy to a single conformer in this region. However, for the purposes of preserving the number of parameters in refinement for a clear comparison, the following discussion will not consider modelling alternative conformations, but switching the predominant conformation modelled. The deposited structure has the same conformation as the molecular-replacement model from which it was derived (PDB entry 1pgb, no X-ray data deposition, not shown). When refined with *REFMAC*5 (Murshudov *et al.*, 2011 ▸), the remodelled structure showed a reduction in the *R*_work_/*R*_free_ gap (16.0/20.2%). Re-running refinement on this structure also reduced the *R*_work_/*R*_free_ gap in *Vagabond* (25.0/25.7%).

To objectively judge whether *Vagabond* was providing a stronger indication of this required correction, the real-space correlation coefficients (Adams *et al.*, 2010 ▸) for the backbone of the tripeptide (residues 9–11) in the corrected and original states were calculated against electron density for maps calculated using both models with sufficient refinement using the chosen software. This was carried out using both *REFMAC*5 and *Vagabond* (the results are summarized in Table 3 ▸). In the case of *REFMAC*5, the correlation coefficient was higher for whichever model was used to generate the input map. Comparing the non-input and input models, this correlation increased from 58.5% to 59.4% against the map generated from the corrected model, but also increased from 56.6% to 57.0% if the noncorrected model was used as the input. These maps were therefore unable to clearly distinguish between the true observation and the calculated model due to bias from the input. On the other hand, *Vagabond* shows a clear favour towards the corrected model regardless of which is used for the input map: if the correct model is used to generate the map, the incorrect model correlation of 53.6% is significantly lower than that of the corrected model, 59.6%. However, contrary to conventional refinement, this preference holds even in the case where the input model is incorrect, as the correlation increases from 53.0% for the incorrect model to 58.0% for the correct model. This shows that *Vagabond* refinement produces maps which are not as strongly biased by the input model.

**Figure 6:**
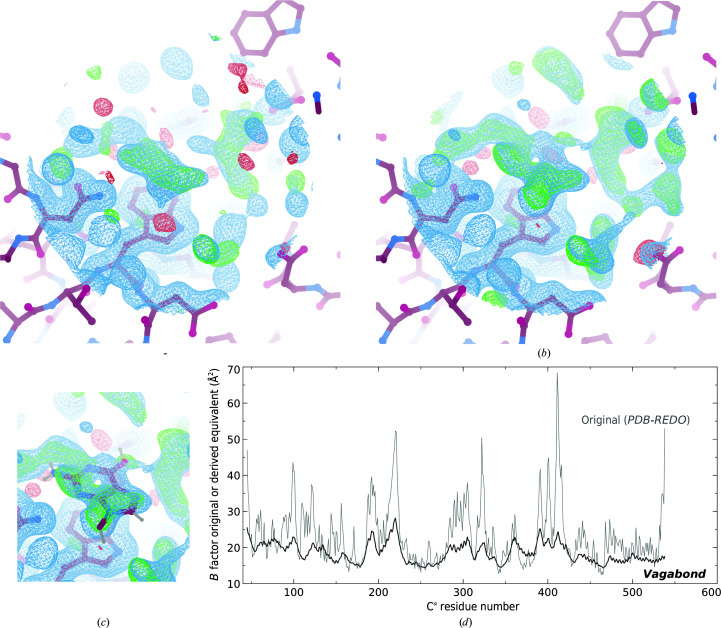
(*a*) For PDB entry 5g5y, averaged weighted density over two copies in the asymmetric unit (*mF*_o_ − *DF*_c_, 1σ), 0.7σ level, and difference density (*F*_o_ − *F*_c_), 2.7σ level, for the *PDB-REDO* structure recalculated without water molecules. (*b*) Refinement with *Vagabond* from the initial PDB file without water molecules; averaged weighted density over two copies in the asymmetric unit (2*F*_o_ − *F*_c_ and phase spread, Section 2), 0.7σ level, and difference density (*F*_o_ − *F*_c_), 2.7σ level. (*c*) Methylguanine placed using *Coot* real-space refinement to demonstrate the fit to density. (*a*), (*b*) and (*c*) were drawn in *Coot* (Emsley *et al.*, 2010 ▸) and *Raster*3*D* (Merritt & Bacon, 1997 ▸). (*d*) Plot showing *B* factors from the original PDB file (grey) and the derived equivalent *B* factors from the *Vagabond* structure (black) per C^α^ atom.

The second example demonstrates the clarification of electron-density features in a binding site achieved using *Vagabond*. The *S. pneumoniae* ABC-transporter protein FusA (PDB entry 5g5y, 1.73 Å resolution; Culurgioni *et al.*, 2017 ▸) was re-refined using *Vagabond* to produce *R* factors of *R*_work_ = 20.6% and *R*_free_ = 22.8%, reducing the *R*_work_/*R*_free_ gap compared with the *PDB-REDO* calculation (*R*_work_ = 15.7%, *R*_free_ = 18.7%). Upon refinement with *Vagabond*, density in the ligand-binding site (Fig. 6 ▸ *a*) was clarified to show a ring-shaped density packed against the aromatic ring of Trp314 (Fig. 6 ▸ *b*). Although this unexpected ligand is not identifiable, the density packed against Trp314 is consistent with a pyrimidine ring (demonstrated by modelling methylguanine; Fig. 6 ▸ *c*). This structure had been assumed to be of the apo state, consistent with the observed domain structure. Other structures (PDB entries 5g62, 5g61, 5g60 and 5g5z) show that cognate ligands can successfully displace this contaminant and cause functional domain shifts, none of which exhibit stacking interactions against Trp314. This suggests that Trp314 has bound to a low-affinity metabolite in an unusual binding mode which does not trigger domain closure. The ability to pinpoint such weak binding events may facilitate the detection of unsuspected druggable binding sites. Once again, the *Vagabond* flexibility model follows that derived from atomistic refinement (Fig. 6 ▸ *d*).

## 4. Conclusions   

This study has revisited the fundamentals of how the structural biology community defines macromolecules. The use of atomistic parametrization to refine crystallographic models has been standard since the introduction of the maximum-likelihood method in the late 1990s (Bricogne & Irwin, 1996 ▸; Murshudov *et al.*, 1996 ▸). Since then, macromolecular refinement has been treated mostly as an optimization problem (Afonine *et al.*, 2012 ▸), and the model definition itself has not been successfully revisited, except for further variations on the theme of atomistic schemes. *Vagabond* now provides an alternative method. There is still considerable scope for improvements, such as ensuring a comprehensive fit of backbone flexibility parameters to electron density, refining the details of the interaction between the protein and the solvent, and finding robust validation metrics for these bond-based structures. Validation is an open area of investigation, as validation metrics tuned to Cartesian refinement are un­suitable for *Vagabond* models. For example, bond length and angle geometry should not be imposed on the average structure, but individual conformers will report near-perfect results. As the benefits of this model are independent of the refinement method, these should, for instance, provide a fundamentally fresh approach to modelling flexibility in cryo-electron microscopy data, where *B* factors are currently biochemically nonsensical (Wlodawer *et al.*, 2017 ▸). The results presented here suggest that the potential impact of this more biochemically relevant parameter space may extend to fields such as structural bioinformatics and molecular dynamics. Often, these fields balance atomic motions against bond geometry within the target function, whereas *Vagabond*, by incorporating bond geometry into the parameter space, aligns it with the most biochemically accessible motions.

## 5. Code availability   

This software is distributed as free, open-source software under the General Public Licence (GPL) version 3, with both a command-line and graphical user interface. *Vagabond* (and the libraries on which it depends) can be installed on Linux or Mac OS X without expense. See https://vagabond.hginn.co.uk for download, a manual, installation instructions and documentation. 

## Figures and Tables

**Table 1:** List of commonly used terms in this text beyond those attributable to the standard description of the chemical structure These are also written in bold throughout Section 2.

Term	Meaning
**Original position**	Coordinate describing an atom position as found in the initial PDB file
**Atom point**	Coordinate at which a delta function describes a single potential position of an atom
**Atom-point cloud**	A set of **atom points** that cumulatively describe all modelled positions of a given atom
**Bond**	A chemical link between two atoms for which various parameters may be defined
**Point-to-point bond**	A link pairing an **atom point** each from one **atom-point cloud** connected by a **bond** (Fig. 1 ▸ *a*)
**Atom group**	A set of atoms, all connected to all other atoms in the set by one or more **bonds**
**Ensemble**	A set of **atom-point clouds** from an **atom group**
**Conformer**	A subset of **atom points** in an **atom group** where all **atom points** are connected to one another by **point-to-point bonds**
**Atom average position**	Coordinate defined as the average of all **atom points** in the **atom-point cloud**
**Anchor atom**	Unique atom within a bonded chain from which all other bonded atoms are calculated
**Upstream bond**	A given **bond** inherits positional and directional information from its **upstream bond**
**Downstream bond**	A **bond** may be the basis of inheritance for one or more downstream bonds
**Sister bond**	Sister bonds are those which share the same upstream bond
**Major atom**	The **major atom** of a bond has an **atom-point cloud** inherited from the upstream bond
**Minor atom**	The **minor atom** of a bond is under direct control by the set of parameters of that **bond**
**Flexless structure**	**Ensemble** for all atoms in a chain calculated without any description of flexibility
**Real-space structure segment**	Calculated electron density for a portion of a structure on a cubic voxel grid
**Asymmetric unit structure**	Special case of a **real-space structure segment** corresponding to the full modelled contents of the asymmetric unit
**Unit-cell grid**	Grid of voxels with appropriate morphology such that the reciprocal-structure factors correspond to the amplitudes of crystal reflections

**Table 2:** List of commonly used symbols in this text * denotes a potentially refinable parameter.

Symbol	Meaning
*J*	Number of **atom points** per **atom-point cloud**
*N*	Number of atoms in bonded chain
…, *n* − 1, *n*, *n* + 1, …	Numbering scheme for sequentially bonded atoms
θ_*n*_	*Bond angle directly dictating the placement of the *n*th **atom-point cloud**
*t*_*n*_	*Mean torsion angle directly dictating the placement of the *n*th **atom-point cloud**
*k*_*n*_	*Magnitude of torsion deviations affecting **atom point** placements for the *n*th **atom-point cloud**
{{\bf a}^{j}_{n}}	*j*th **atom point** of the **atom-point cloud** of the *n*th atom
〈**a**_*n*_〉	**Atom average position** of *n*th atom; = \textstyle \sum_{j = 1}^{J}({\bf a}^{j}_{n})/J
{{\bf b}^{j}_{n}}	Vector describing **point-to-point bonds** connecting the (*n* − 1)th **atom point** to the *n*th **atom point**; = ({\bf a}^{j}_{n-1}-{\bf a}^{j}_{n})
〈**b**_*n*_〉	Average bond vector connecting the (*n* − 1)th **atom average position** to the *n*th **atom average position**; = \textstyle \sum_{j = 1}^{J}({\bf b}^{j}_{n})/J
{\Delta {\bf a}^{j}_{n}}	Displacement of the *j*th **atom point** from the **atom average position** for atom *n*; = ({P\bf a}^{j}_{n}-\langle {\bf a}_{n}\rangle)
*P*_*n*_	Plane intersecting 〈**a**_*n*_〉 and parallel to 〈**b**_*n*_〉 and 〈**b**_*n*−1_〉
{\hat{\bf p}_{n}}	Unit vector perpendicular to *P*_*n*_
α_*n*_, β_*n*_	**Two angles used to form a rotation matrix associated with the *n*th atom
**M**_*n*_	Rotation matrix **M**_*n*_ defined in terms of β_*n*_ and α_*n*_ as in (5)
s^{j}_{n}	Sine of the angle between {{\bf M}_{n}}{\hat{\bf p}_{n}} and {\Delta {\bf a}^{j}_{n}} for the *j*th **atom point** of the *n*th atom
\Delta t^{j}_{n}	Torsion-angle deviation for the **point-to-point bond** associated with the *j*th **atom point** for the *n*th atom; = (k_{n}s^{j}_{n})
**T**	Symmetrical tensor describing translational offsets of **atom points** for the **anchor atom**
**r**	Vector used to calculate rotations for individual **atom points** for the **anchor atom**
**w**	Vector used to calculate rotation-dependent translations for individual **atom points** for the **anchor atom**
\Delta{{\bf a}^{j}_{T}}	The partial positional offset from the **average atom position** of anchor **atom point** *j*, calculated from **T** and {{\bf a}^{j}_{n}}
\Delta{{\bf a}^{j}_{S}}	The positional offset from the **average atom position** of anchor **atom point** *j*, calculated from \Delta{{\bf a}^{j}_{T}} and each pair of **r** and **w** parameters.
*d*_*j*_	Dot product between translational offset for **anchor atom** and rotation vector **r**; = \Delta{{\bf a}^{j}_{T}} · **r**
*k*	Reflection at a given Miller index
*s*_*k*_	Standard error for reflection *k*, = σ*F*_o_/*F*_o_
*d*_*k*_	Downweighting value for reflection *k*; = \exp(-s_{k}^{2})
\sigma_{\varphi}^{k}	Standard distribution of phase angles for reflection *k*

**Table 3:** Comparison of real-space correlation coefficients for the analysis of PDB entry 2on8 in the text using main-chain atoms for the tripeptide residues 9–11 Atomistic parametrization was provided by *REFMAC*5 and bond-based parametrization by *Vagabond*.

	Atomistic		Bond-based	
	Map calculated with original model	Map calculated with corrected model	Map calculated with original model	Map calculated with corrected model
Corrected model	56.6%	59.4%	58.0%	59.6%
Original model	57.0%	58.5%	53.0%	53.6%
